# Investigating the influence of titanium particle size and concentration on osteogenic response of human osteoblasts – in vitro study

**DOI:** 10.2340/biid.v11.40843

**Published:** 2024-06-13

**Authors:** Soumya Sheela, Waad Kheder, A B Rani Samsudin

**Affiliations:** a Research Institute for Medical and Health Sciences, University of Sharjah, Sharjah, United Arab Emirates; b College of Dental Medicine, University of Sharjah, Sharjah, United Arab Emirates

**Keywords:** Titanium dioxide, osteoblasts, particles

## Abstract

**Purpose:**

The purpose of this study was to investigate the correlation between the size and concentration of titanium particles and the osteogenic response of human osteoblasts (HOB).

**Materials and Methods:**

Different concentrations of titanium dioxide nano- and micro-particles were prepared and their biocompatibility on HOBs was analyzed using XTT assay. The changes in the actin cytoskeletal organization were studied by confocal laser scanning microscopy. The generation of intracellular reactive oxygen species (ROS) by HOBs after exposure to titanium dioxide particles was analyzed using ROS assay. Besides, the osteogenic potential represented by alkaline phosphatase activity, osteoprotegerin, macrophage colony stimulating factor levels, and biomineralization were analyzed.

**Results:**

Short-term interaction of titanium dioxide nano- and micro-particles did not induce toxicity to HOBs. However, cells treated with 100 μg/mL titanium dioxide nano- and micro-particles demonstrated higher ROS generation compared to control. Besides, cells treated with 100 μg/mL titanium dioxide nanoparticles showed higher alkaline phosphatase activity, osteoprotegerin, macrophage colony stimulating factor levels and biomineralization compared to titanium dioxide microparticles.

**Conclusion:**

Collectively, the study found titanium dioxide nanoparticles to be more biocompatible than microparticles providing an insight into the capability of nanostructures in supporting osteoblast differentiation and its plausibility in biomedical applications.

## Introduction

The bone remodeling process depends on osteoblast and osteoclast activity. This process activity is often challenged by the presence of foreign bodies leached from orthopedic or dental prostheses [1]. These devices are made of biocompatible materials that should not provoke any cellular harm to the peri-implant environment. The most successful biomaterial used in implanted prosthetic devices is titanium alloy due to its biocompatibility, strength, and corrosion resistance properties. However, the implanted prosthesis in the oral cavity environment is subjected to corrosion as its micromotion during function may result in the production of wear particles in the peri-implant osseous tissue microenvironment [2]. Implant wear particles may even be produced earlier during the surgical implantation stage of the implant due to frictional contact between the prosthesis and the osseous channel wall leading to the generation of micro- and nano-size titanium particles in the peri-implant microenvironment. Costa et al. demonstrated the fate of wear particles penetrating osteoblast cell membranes and the internalization by other living cells [3]. This exposure to wear particles will certainly have an impact on the functionality of osteoblasts and other cells of the immune system.

Cells of the osteoblast lineage support two apparently distinct bone regeneration functions, namely bone deposition and promotion of osteoclast formation [4]. Osteoblast phenotypes that support osteoclast differentiation and bone formation are still not well understood, particularly when the remodeling environment involves the presence of an implanted titanium prosthesis. Leaching of titanium dioxide particles from the implant surface has been shown to further compound the healing capacity due to the complexity of peri-implant cellular responses [5]. Dental practitioners are unaware of the leaching activity of implants following exposure to the implant surface, the use of acidic dental fluoride dentifrices, and the action of microorganisms on the titanium surfaces. Since these activities occur at the subclinical level, the pathology will propagate to end with the development of peri-implantitis.

The leached titanium particles have an intricate effect on the production of reactive oxygen species (ROS). ROS is not only essential for normal cellular metabolism and signal transductions but may also induce cell death, and damage of DNA, RNA, and protein when it is excessive [6]. Kheder et al. demonstrated the impact of the size and concentration of titanium dioxide nanoparticles (TiO_2_NPs) and microparticles (TiO_2_MPs) on macrophage production of intracellular ROS in vitro [7]. Besides, upon cellular uptake and internalization, the titanium dioxide nanoparticles enhance the production of superoxide anion and thereby alter the antioxidant mechanism in human osteoblasts (HOB) [8].

It is very well documented that these wear particles stimulate the release of proinflammatory cytokines and chemokines, which in turn inhibit the osteoblastic activity and favor osteoclastogenesis [9]. However, the effect of these wear particles on the osteoblasts based on their concentration, size, and morphology is not well demonstrated. Studies have shown that TiO_2_ nanoparticles have varying effects on osteoblasts and osteoblast-like cells [10, 11]. Osteogenic inhibition and bone loss induced by the wear of titanium alloy are crucial in the instigation of peri-prosthetic osteolysis [12]. Nevertheless, the underlying molecular mechanism behind this phenomenon is poorly understood.

Titanium oxides are found to enhance alkaline phosphatase (ALP) production in human fetal osteoblasts and are one of the key bioactive elements responsible for the excellent biocompatibility of titanium alloys [13]. Osteoblast/Osteoclast maturation and resulting bone remodeling are indeed the effects of many molecules, some of which are Osteoprotegrin (OPG), RANK/RANKL, and Macrophage colony-stimulating factor (M-CSF) [14, 15]. OPG is secreted by osteoblasts and inhibits excessive bone resorption by competing and preventing RANKL from binding to RANK receptors on the osteoclasts [16]. The OPG/RANKL ratio is an important factor in determining the bone mass in normal and osteoporotic cases [14]. Previous studies have demonstrated that TiO_2_ particles can disrupt the OPG/RANKL homeostasis and inhibit the osteoblasts' activity [11]. M-CSF is another secretory cytokine that plays a crucial role in maintaining the osteoblast/osteoclast equilibrium [17]. Though many studies have investigated the impact of titanium dioxide particles on the HOB cells, little is still known about the effect of sizes and concentrations of TiO_2_ particles on the differentiation of HOBs and their expression of OPG and M-CSF.

Despite adherence to the standard surgical protocol and oral care, the development of peri-implantitis that ends with the failure of implant osseointegration still occurs. Many dentists are not informed about recent updates in the literature regarding the preventive measures to be taken to minimize the leaching of particles from implant surfaces. Besides, little is known about the role of these particles in the failure of osseointegration. The purpose of this study was to investigate the correlation between the size and concentration of titanium particles and the osteogenic response of HOBs that modulate peri-implant bone remodeling.

## Materials and methods

### HOB cell culture

HOB cells were procured from AddexBio (AddexBio P0004010, San Diego, CA, USA) and were maintained in DMEM/F-12 cell culture medium (Gibco, Thermo Fisher Scientific, Waltham, MA, USA) supplemented with 10% fetal bovine serum (FBS) and 1% penicillin-streptomycin (Sigma-Aldrich, St. Louis, MO, USA) in a CO_2_ incubator with 5% CO_2_. The medium was changed every 2–3 days and the cells were subcultured after attaining 70–80% confluency. The cells between three and six passages were used for all the experiments.

### Titanium dioxide (TiO_2_) particles preparation and characterization

The TiO_2_ nanoparticles (TiO_2_NPs) and microparticles (TiO_2_MPs) were prepared following a protocol earlier reported [7]. Briefly, TiO_2_NPs and MPs having a particle size of < 100 nm and < 5 μm in diameter procured from Sigma (Sigma-Aldrich, St. Louis, MO, USA) were used for the study. A total of 10 mg of TiO_2_NPs and MPs were weighed separately and dispersed in 10 mL deionized water in a sonicator (Qsonica sonicators, USA) operated at 40% in pulse mode (50s on /50s off) for 10 min. The hydrodynamic diameter, the poly dispersity index (PDI) and the zeta potential of TiO_2_NPs and MPs were measured using a Malvern, Zetasizer Nano-ZS system (Malvern Instruments, UK). The measurements were done at 25°C using a particle concentration of 10 μg/mL for NPs and 50 μg/mL for MPs. For the cell culture studies, 1 mg/mL stock suspension of both TiO_2_NPs and MPs were prepared in complete DMEM/F-12 cell culture medium and 5, 20, and 100 μg/mL working concentrations prepared from the stock for cell treatments.

### Cell viability assay on HOBs

To determine the effect of TiO_2_ particles on the viability of HOB cells, an XTT (Cell Proliferation Kit II (XTT), Sigma-Aldrich, USA) assay was performed. HOB cells at a density of 1 × 10^4^ cells/well were seeded onto 96-well cell culture-treated plates in a final volume of 100 μL of complete DMEM/F-12 culture medium. The cells were treated for 24 h with different concentrations of TiO_2_ nanoparticles and microparticles (100, 70, 50, 20, 10, and 5 μg/mL). Cells without TiO_2_ treatment were taken as the experimental control. After particle treatment, 50 μL of the XTT was added to each well and incubated for 4 h in a CO_2_ incubator as per the manufacturer’s instructions. The absorbance was then quantified using a plate reader (Synergy H1 Microplate Reader, Biotek Instruments, Winooski, VT, USA) at 450 nm and reference wavelength of 630 nm. Care was taken to ensure the TiO_2_ particles used in the experiments were endotoxin-free.

### Total ROS assay

For the intracellular ROS quantification, HOBs at a density of 200,000 cells/well were seeded in a 6-well cell culture treated plate, followed by three different concentrations of TiO_2_NPs and MPs (5 μg/mL, 20 μg/mL, and 100 μg/mL) and treated for 24 h. The treated cells were then collected by trypsinization, and the cell pellet was incubated with 25-μM ROS dye (DCFDA / H2DCFDA – Cellular ROS Assay Kit, Abcam, Cambridge, UK) for 30 min in the dark at 37°C. The cells were then washed with 1X Buffer and the cell suspension was transferred to a 96-well black microplate with a clear bottom and the fluorescent intensity was measured using a fluorescence microplate reader (Synergy H1 Microplate Reader, Biotek Instruments, USA) at an excitation/emission wavelength of 485 nm and 535 nm, respectively. The percentage ROS production was calculated with respect to the HOBs without TiO_2_ treatment.

### Actin cytoskeleton staining

The effect of different concentrations of TiO_2_NPs and MPs (5, 20, and 100 μg/mL) on the cytoskeletal morphology of HOBs was analyzed after exposing the cells to the particles for 48 h. After the incubation time, the cells from each group were subjected to cytoskeleton staining. Briefly, the cells were fixed with 4% paraformaldehyde (PFA) for 15–20 min at room temperature and permeabilized with 0.1% Triton X-100 solution (Sigma-Aldrich) for 5 min at room temperature. Subsequently, the cells were incubated with Texas Red™-X Phalloidin (Thermo Fisher Scientific, USA) for 20 min at room temperature, washed with PBS, and the nucleus was counterstained with DAPI staining (Abcam, Cambridge, UK) for around 5 min at room temperature. Changes in the cytoskeleton arrangement were measured by a Confocal Laser Scanning Microscopy (Nikon Eclipse Ti Elements, Nikon Instruments Inc., Japan) with respect to the untreated HOBs.

### Alkaline phosphatase (ALP) activity

The alkaline phosphatase activity of the HOBs treated with TiO_2_NPs and MPs was analyzed by using the ALP kit (Abcam, Cambridge, UK). The HOBs were seeded at a density of 50,000 cells per well onto a six-well plate in DMEM/F-12 NM. Once the cells attained 70–80% confluency, the medium was changed to an osteogenic medium (OM), which is basically DMEM/F-12 normal cell culture medium supplemented with 10^-8^ M dexamethasone (Sigma-Aldrich, USA), 10 mM β-glycerophosphate (Sigma-Aldrich, USA) and 50 μg/mL L-Ascorbic acid 2-phosphate (Sigma-Aldrich, USA). The cells were further treated with two different concentrations of TiO_2_NPs and MPs (20 and 100 μg/mL) for a period of 14 days in both NM and OM. The medium was changed every 3 days keeping the concentration of particles constant. The cells grown in NM and OM at day 7 and after 14 days were checked for the ALP activity. Briefly, the cells were trypsinized to get the cell pellet vortexed and further sonicated in a probe sonicator and the supernatant was collected after centrifugation at 12,000 g at 4°C for 15 min. The collected supernatant was further analyzed for the ALP activity. As per the manufacturer’s instructions, 50 μL of 5mM p-nitrophenyl phosphate (pNPP) substrate was added to the supernatant, and the plates were incubated at 25°C for 60 min in the dark. The optical density of the colored p-Nitrophenol (pNP) formed by the conversion of pNPP substrate by the ALP present in the supernatant was measured at 405 nm using a microplate reader (Synergy H1 Microplate reader, Biotek Instruments, USA) after stopping the reaction with 20 μL of stop solution. Untreated HOBs in NM were considered as the experimental control.

### Enzyme-linked immunosorbent assay

TiO_2_-treated HOBs cultured for 7 and 14 days in both OM and NM were later examined for analysis of the cytokine levels. M-CSF and OPG levels secreted by the particle-treated HOB cells in the culture supernatants were measured using enzyme-linked immunosorbent assay (ELISA) kits (Abcam, Cambridge, UK) following the manufacturer’s instructions. Briefly, the collected culture supernatant was added to anti-M-CSF and OPG antibody-coated wells along with an antibody cocktail solution and were kept for 1 h of incubation at 37°C. Following this, the wells were washed three times with 1X wash buffer PT and 100 μL of TMB development solution was added and the plates were kept for 10 min incubation in the dark with gentle shaking. The absorbance was measured at 450 nm using a microplate reader (Synergy H1 Microplate reader, Biotek Instruments, USA) after stopping the reaction by adding 100 μL of stop solution. The concentrations of M-CSF and OPG in the culture supernatant were calculated from the M-CSF and OPG standard curves. Untreated HOBs in NM were considered as the experimental control.

### Calcium mineral deposition – Alizarin Red S assay quantification

For analyzing the mineralization capacity of the HOBs treated with TiO_2_NPs and MPs, cells at passage three were seeded onto a six-well plate at a density of 50,000/well and maintained in DMEM/F-12 cell culture media (NM). The HOBs were treated with TiO_2_NPs and MPs and were cultured for a period of 14 days in both OM and NM. Media were replaced every 3 days. At each time point (7 and 14 days), the plates with the cells were processed by fixing with 4% PFA for 15–20 min at room temperature. After washing the plates with deionised water, 40 mM Alizarin Red S solution (pH 4.1, Sigma-Aldrich, USA) was added to each well and kept for 20–30 min at room temperature with gentle shaking. The cells were washed well with deionized water to remove excess dye and the stained calcium nodules were captured using an Olympus IX53 Inverted Microscope (Olympus Corporation, Tokyo, Japan). Calcium quantification was next performed by extracting the dye from the stained mineral deposits by using a 10% (v/v) acetic acid protocol as previously described [18]. The absorbance of the eluted stain was read at 405 nm in a microplate reader (Synergy H1 Microplate reader, Biotek Instruments, USA). Untreated HOBs in NM were considered as the experimental control.

### Statistical analysis

One-way ANOVA followed by Dunnett’s multiple comparisons test was used to analyze the results, applying a 0.05 level of significance. The statistical analysis was performed using GraphPad Prism version 9.0.0 (121) software (GraphPad Software, Inc., San Diego, CA, USA). All experiments were repeated three times (*n* = 3) with six technical replicates.

## Results

### TiO_2_NPs and MPs size and viability study

**Figure 1:**
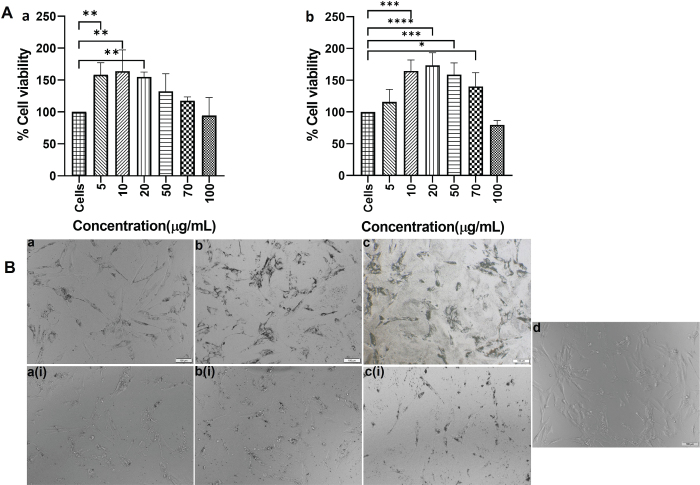
A) XTT cell viability assay of human osteoblasts (HOB) cells after treatment with TiO_2_ NPs (a) and MPs (b) for a period of 24h. Data are represented as mean ± standard deviation. Statistical significance was observed among the different concentrations of TiO_2_NP, and MP administered mainly at lower concentrations when compared to non-treated cells. Significance was represented with *, **, *** and **** which represents a *p*-value <0.5, 0.01, 0.001 and 0.0001, respectively. B) Representative phase contrast microscopic images of the TiO_2_NPs and MPs treated HOB cells. The upper panel (a, b, c) represents the treatments with 5 μg/mL, 20 μg/mL, and 100 μg/mL TiO_2_NPs treatment and the lower panel (a(i), b(i), c(i) represents the TiO_2_MPs treatment. The morphological changes after treatment were compared with that of control HOB cells (d).

The DLS measurements of TiO_2_NPs gave a mean diameter of 330.467 ± 43.127 nm, zeta potential value −7.857 ± 0.276 mV, and PDI of 0.369 ± 0.063. The TiO_2_MPs showed a diameter of 2525.333 ± 1174.277 nm with −8.637 ± 0.605 mV as the zeta potential, and 0.594 ± 0.073 as the PDI. The XTT assay results showed that both TiO_2_NPs and MPs at all concentrations tested were non-cytotoxic to HOBs (Figure 1A). Statistical significance differences in the viability were observed among the different concentrations of TiO_2_NPs and MPs administered, mainly at lower concentrations when compared to non-treated cells. The non-toxic behavior of TiO_2_NPs and MPs at all the tested concentrations was evident from the phase contrast microscopic image as well (Figure 1B). Unlike the lower concentrations, the cells treated with 100 μg/mL TiO_2_NPs, and MPs showed lower cell density, although the difference was not statistically significant.

### Morphological and cytoskeletal integrity

**Figure 2:**
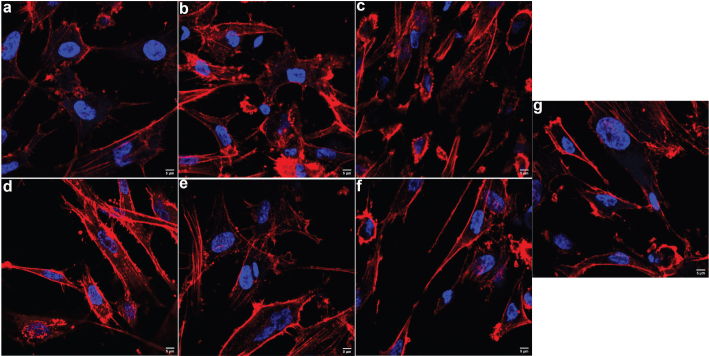
Representative confocal microscopic images showing the cytoskeletal organization of human osteoblast (HOB) cells after being treated with different concentrations and size of TiO_2_ particles after 48h of incubation. Upper panel was HOBs treated with 5, 20 and 100 μg/mL (a, b, c, respectively) of TiO_2_NPs and lower panel was HOB treated with 5, 20 and 100 μg/mL (d, e, f, respectively) of TiO_2_MPs. There was no alteration in the cytoskeletal arrangement among the treated HOBs and their post-treatment morphology after 48 h was comparable to that of the non-treated control HOB cells (g). Texas red phalloidin was used for actin staining and DAPI (blue) for nuclei. The images were captured under 60X objective.

Three different concentrations (5, 20, and 100 μg/mL) of TiO_2_NPs and MPs were used in the treatment of HOBs. None of the tested concentrations disrupted the cytoskeletal arrangement of HOBs and were comparable to the untreated HOBs (Figure 2).

### Generation of ROS

**Figure 3:**
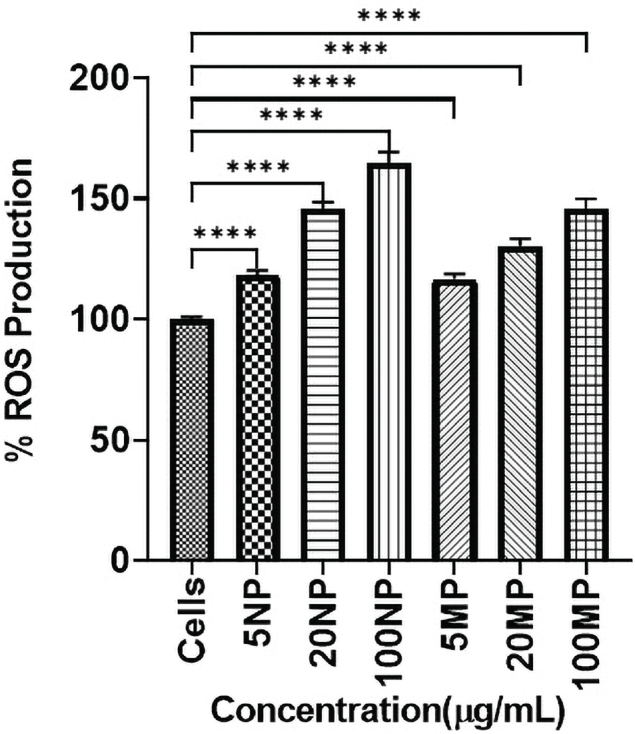
Bar chart representing the percentage of Reactive Oxygen Species (ROS) generation (mean ± SD) of each human osteoblast (HOB) cell treated with titanium dioxide nanoparticles (TiO_2_NPs) and microparticles (MPs) with respect to control HOB (Cells). Statistical significance is represented as **** which signifies the *p*-values < 0.0001.

A significant (*p* < 0.05) increase in ROS production was observed at all three concentrations of TiO_2_NPs and MPs compared to the untreated control (Figure 3). The maximum ROS activity was observed for HOBs treated with 100 μg/mL NPs and 100 μg/mL MPs TiO_2_. Overall, TiO_2_NPs showed more ROS generation than TiO_2_MPs.

### Alkaline phosphatase activity

**Figure 4:**
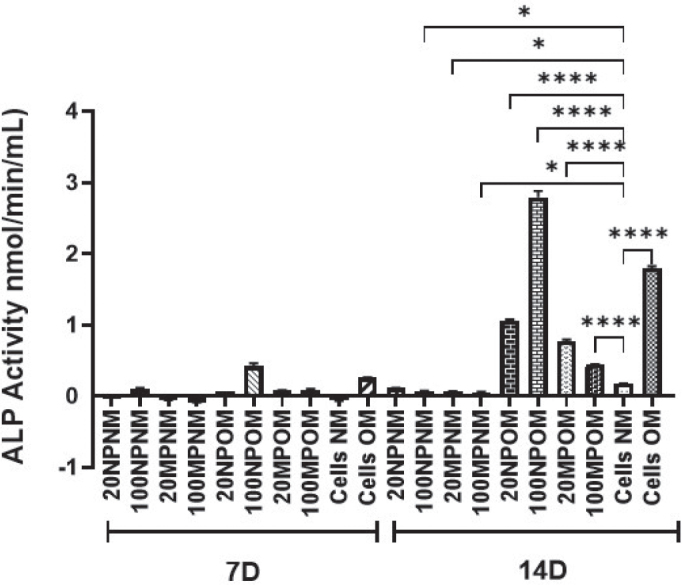
Alkaline phosphatase (ALP) activity of human osteoblasts (HOBs) after 7 and 14 d of treatment with titanium dioxide nanoparticles (TiO_2_NPs) and microparticles (MPs) in normal media (NM) and osteogenic media (OM). Increase in ALP activity was observed at 14 d of culture conditions only. Statistically significant differences were observed in the ALP activity of TiO_2_ treated HOBs in OM when compared with untreated cells grown in NM. Those cells treated with 100 μg/mL NPs in OM (100NPOM) demonstrated a high ALP activity at 14 d compared to TiO_2_MPs and untreated cells in NM and OM. Cells treated with 100 μg/mL MPs in OM (100MPOM) showed a lower ALP activity compared to those treated with NPs and untreated HOB cells cultured in OM. Significance was represented with * and **** which represents a *p*-value <0.5 and 0.0001, respectively.

At day 7, HOBs exposed to 20 and 100 μg/mL of TiO_2_NPs and MPs in NM demonstrated a very slow osteogenic differentiation represented by low alkaline phosphatase activity, and the level remained unchanged until day 14. However, HOB exposed to 20 and 100 μg/mL of TiO_2_NPs and MPs in OM demonstrated an initially low osteogenic activity with low alkaline phosphatase level on day 7, which subsequently rose to higher levels by day 14 compared to the control cells (Figure 4). HOBs exposed to 100 μg/mL of TiO_2_NPs demonstrated a significant increase in ALP activity.

### OPG and M-CSF expression

**Figure 5:**
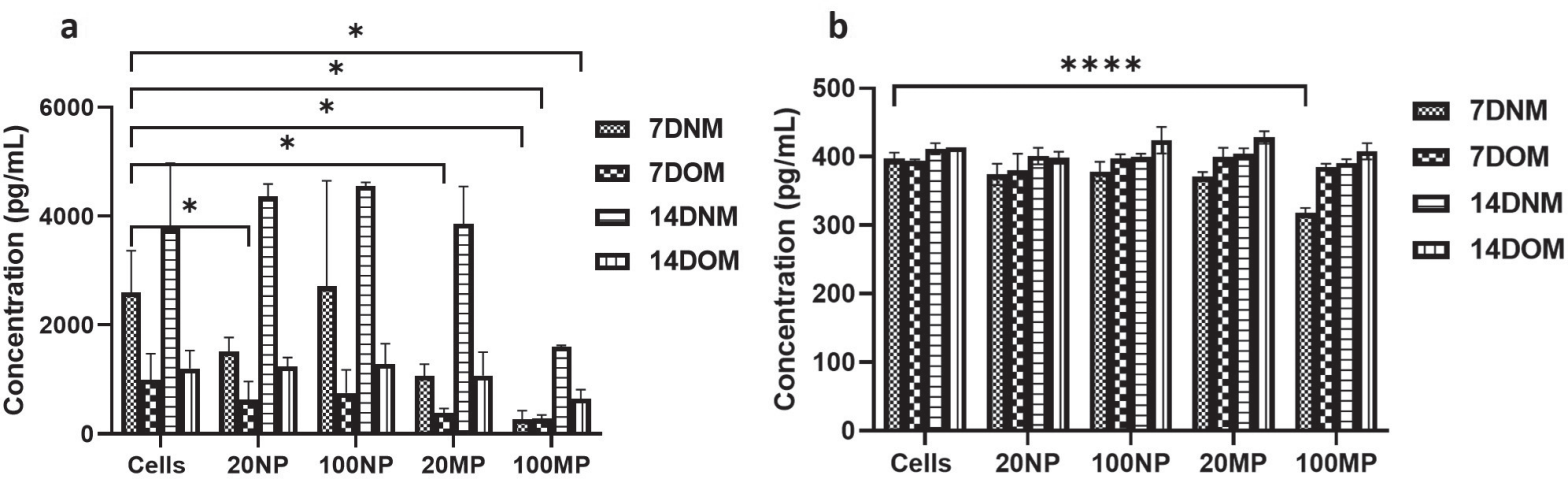
Expression of (a) osteoprotegerin (OPG) and (b) macrophage-colony stimulating factor (M-CSF) in human osteoblast (HOB) after 7 and 14 d of treatment with 20 and 100 μg/mL of titanium dioxide nanoparticles (NPs) and microparticles (MPs) in normal media (NM) and osteogenic media (OM); using ELISA. Data are represented as mean ± SD from three separate experiments. The comparison is made between the tested HOBs and untreated cells in NM and OM (Cells). Statistical significance is denoted with * and ****, which represents the *p*-values <0.5 and 0.0001, respectively.

There was a decrease in the production of OPG after treatment of HOBs with 20 and 100 μg/mL TiO_2_NPs and MPs in OM for 7 or 14 days compared to NM for 7 and 14 days. HOBs treated with 100 μg/mL TiO_2_MPs showed less production of OPG in comparison to 100 μg/mL TiO_2_ NPs or non-treated HOBs. The expression after treatment with 20 and 100 μg/mL TiO_2_NPs was like that of the cell control after both 7 and 14 days, where an increase in OPG level was observed especially in 14 days (Figure 5a). There was no significant change in the level of M-CSF secretion when HOBs were exposed to both TiO_2_NPs and MPs at all concentrations in normal and osteogenic media throughout the 14 days. However, 100 μg/mL TiO_2_MPs cultured in NM for 7 days showed a much lower M-CSF production compared to the control (Figure 5b).

### HOB mineralization – Alizarin red staining (ARS)

**Figure 6:**
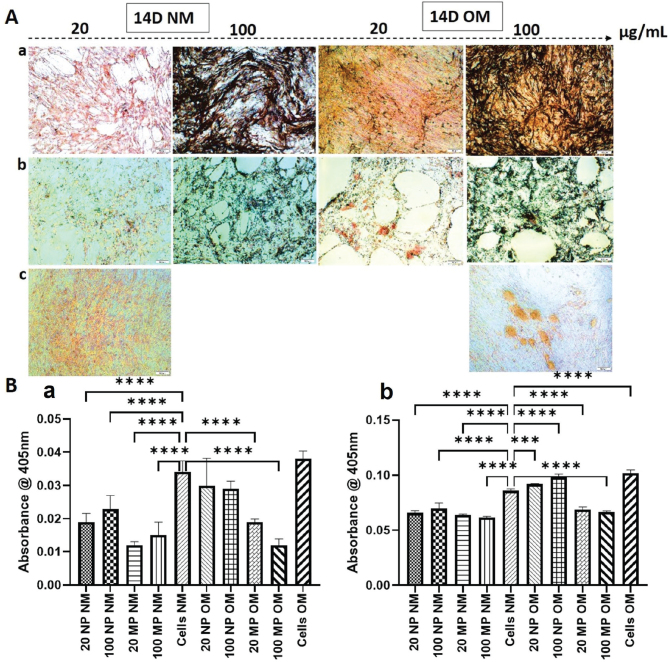
(A) Representative images of human osteoblast (HOB) cells stained with Alizarin Red S after 14 d of treatment with TiO_2_ nanoparticles (TiO_2_NPs) (panel a) and TiO_2_ microparticles (TiO_2_MP) (panel b) and untreated control HOB (Cells) (panel c) in normal media (NM) and osteogenic media (OM). As evident from the images, higher amount of calcium deposits was seen on untreated cells as well as in HOB treated with100 μg/mL of titanium dioxide nanoparticles in OM (100 NPOM). (B) represents the quantification of mineralized nodules formed in the wells after 7 d (a) and 14 d (b) of culture. The results are represented as mean ± standard deviation from three independent experiments done in triplicates. **** represents *p*-value < 0.0001.

Generally, 14 days of treatment of HOB with TiO_2_ resulted in more calcium deposition than did 7 days of treatment. The level of calcium deposition by HOBs treated with 20 and 100 μg/mL TiO_2_NPs and MPs in NM was less than that of non-treated cells in NM. However, the highest level of deposition occurred following treatment of HOBs with 20 and 100 μg/mL TiO_2_NPs in OM, and for 100 μg/mL TiO_2_NPs the calcium deposition was almost like non-treated cells in OM (Figure 6A and 6B).

## Discussion

In dental clinical practice, higher concentrations of titanium particles were detected in mucosal tissue obtained from peri-implantitis sites compared to the tissue from clinically healthy peri-implant sites [19]. These findings suggest that titanium particles and ions are leached into the peri-implant mucosal tissues and that tribocorrosion may play a role in the pathogenesis of peri-implant diseases. Biological complications of dental implants associated with titanium particle release are always accompanied by an intense chronic inflammatory response that is resistant to routine clinical therapeutic measures and prevention [20]. The involvement of bone building and bone resorbing proteins in the peri-implant microenvironment in such conditions is complex and demands further elucidation.

In this study, the viability assay showed that no cytotoxicity was observed after 24 h of HOBs exposure to TiO_2_NPs and MPs. These observations agree with previous findings [21, 22] that at lower concentrations for shorter time periods, there is slight increase in the cell viability.

Under the experimental conditions of this in vitro study, the generation of ROS occurred when HOBs were exposed to TiO_2_ particles at non-cytotoxic concentrations. The amount of ROS generated was concentration-dependent whereby 100 μg/mL of both TiO_2_NPs and MPs produced the highest amount of ROS. Besides, TiO_2_NPs generated significantly more ROS than MPs, which invariably leads to higher proinflammatory cytokine release [23]. However, Kheder et al., found that particle concentration does determine the degree of cellular oxidative stress [7] and the role of particles size is however subjected to controversy [24].

Furthermore, the results from other authors are not consistent due to the differences in particle characteristics such as crystallography, shape and particles aggregation that have considerable effects on oxidative stress generation [21]. Despite those factors, NPs are more prone to cell uptake by simply internalizing into the cell through the plasma membrane and even entering the nucleus through nuclear membrane pores as a result of their nano size [25]. This study showed a time-dependent HOBs viability following exposure to TiO_2_MPs at all concentrations compared to TiO_2_NPs suggesting an unfavorable immediate effect when HOBs are exposed to large-size particles. However, other studies have also shown that TiO_2_NPs may cause more toxic effects in the long term [26, 27]. There is strong recent evidence that NPs’ properties such as size, chemical surface, charge, and topography influence cell behavior, and mediate various molecular processes for the regulation of cellular functions [28].

Cytoskeleton disruption with disturbance of cell-matrix interactions may be due to oxidative stress or direct cell-foreign particle interaction. Oxidants may selectively cause damage to DNA, lipids, and proteins in cells with resulting changes in cytoskeleton organization and dynamics [29]. In this study, confocal microscopy of HOBs did not demonstrate any modifications of the actin cytoskeleton even following exposure to the highest concentration of TiO_2_NPs and MPs. This may indicate the ability of HOBs to protect and modulate their cytoskeleton dynamics at the non-cytotoxic TiO_2_ concentrations tested. In other situations, oxidative stress may disrupt the actin cytoskeleton and could provoke the formation of oxidation-induced actin bodies (OABs) [30]. These features were not observed in this study and the HOBs exposed to TiO_2_ particles remained morphologically healthy. Confocal microscopy in this study further confirmed good adhesion strength and spreading and intact cell membranes.

By day 7, the presence of TiO_2_NPs and MPs in NM delayed the differentiation potential of osteoblasts, releasing very low ALP while at the same time, OPG secretion was higher, suggesting an attempt to downregulate osteoclastogenesis. Both their levels remained the same until day 14. Moreover, the level of M-CSF did not change, indicating any attempt to proceed toward osteoclast maturation. The results also suggest the inability of HOBs to curb oxidative stress following exposure to TiO_2_ particles in normal media as another likely cause of the poor osteogenic activity. Since this phenomenon will lead to lower bone regeneration capacity, HOBs would release a higher amount of OPG that could block the recruitment of monocytes into future osteoclasts and allow prevention of bone-resorbing activity. Other authors have observed similar phenomena and concluded that the adverse effects of TiO_2_ particles on osteoblast differentiation and bone destruction are facilitated by the GSK-3β/β-catenin signal pathway [12]. As mentioned earlier, the proliferation and differentiation of HOBs are influenced by ROS production in response to titanium particles. In this study, HOBs in OM showed an initially low ALP level on day 7. However, the presence of the osteogenic induction agent within the media was able to enhance ALP to higher levels by day 14 compared to the control cells.

When HOBs without TiO_2_ treatment were grown in OM, there was a much higher spike in ALP activity by day 14. However, there was a significant reduction in the ALP level following treatment with 100 TiO_2_MP in OM. The findings showed an increase in the ALP level in response to 100 NP TiO_2_ particles compared to control osteogenic media. On the other hand, the reduced ALP level in 100 MP TiO_2_ when compared to OM, is indicative of the influence of particle size on the ALP level. Osteogenic media support the scavenging of ROS and free radicals and propagate the differentiation of osteoblasts by demonstrating higher ALP activity. Osteogenic media contain antioxidant properties that maintain cellular genomic stability, promote cell adhesion to the extracellular matrix, and support osteoblast differentiation and bone formation by regulating mitochondrial stress [31, 32].

HOBs grown in NM without TiO_2_ treatment showed upregulation of OPG on day 14. This high amount of OPG would inhibit further osteoclast differentiation and allow low bone resorption. On the other hand, the treatment of HOBs with TiO_2_NPs also showed upregulation of OPG similar to that of the control cells. However, after both 7 and 14 days, the TiO_2_MPs showed downregulation of OPG in comparison to control cells. These findings confirm the role of particle size in the expression of osteoclast osteogenic factors by HOBs. The downregulation in the OPG was significant in the 100μg/mL TiO_2_MPs treated groups.

In this study, there were minimal changes in the level of M-CSF secreted by HOBs grown in both normal and osteogenic media, irrespective of TiO_2_ treatment throughout the 7 or 14 days, and their levels were in the similar range with the control groups. However, the level of M-CSF was downregulated following seven exposures of HOBs to 100μg/mL TiO_2_MPs. It is a known fact that M-CSF levels are related to the differentiation state of HOBs [33]. Hence, in our findings, it was obvious that 100μg/mL TiO_2_MPs is unfavorable for the differentiation of HOBs. In contrast, other authors have observed that titanium induces the production of chemokines including M-CSF in HOBs [34]. In this study, the downregulation in ALP expression is associated with the downregulation of OPG and M-CSF levels by HOBs following treatment with 100 μg/mL TiO_2_MPs. Other authors have demonstrated the reverse relationship between ALP and OPG in osteoblast cells in vitro experiments related to the generation of ROS and acidosis [35]. M-CSF is secreted by osteoblasts and its precursor cells; however, osteocytes contribute to most of the M-CSF secretion. M-CSF is usually found in two distinct forms: a membrane-bound and a secreted form. This study investigated the secreted form which could be low in the supernatant and not detected under the experimental condition [36]. M-CSF is a crucial factor responsible for the differentiation of pre-osteoclasts to osteoclasts. Nonetheless, M-CSF on its own is unable to enhance this process without additional co-stimulatory signals such as RANKL, as demonstrated by Starlinger et al. in an animal model study [37]. A rise in the level of M-CSF secretion is expected when HOBs cultivated in normal media are exposed to TiO_2_ particles with resulting oxidative stress, promoting osteoclastogenesis and bone resorption in vivo [38]. Instead, the results of the present experiment showed that the level was unchanged, suggesting that other associated mechanisms may be involved for M-CSF level such as the degree of the cellular insult as well as the duration of HOBs exposure to TiO_2_ particles.

Mineralization of the extracellular matrix denotes the terminal marker of osteoblast differentiation. In this study, the mineralization assay showed that HOBs exposed to both TiO_2_NPs and MPs at all concentrations in both normal and osteogenic media were able to form mineralized nodules in the extracellular matrix. However, the formed mineralization density was lower compared to unexposed control cells. Meanwhile, HOBs treated with 100μg/mL TiO_2_NPs grown in OM showed mineralization density that was close to that of control. Matrix mineralization density was more intense in untreated HOBs in the control group. This observation suggests a negative influence of TiO_2_NPs and MPs and oxidative stress on osteoblast terminal differentiation [39], and expression of osteoclastogenesis factors [40]. The results of this study suggested that despite stressful treatment with TiO_2_NPs and MPs, HOBs have the capacity to repair and maintain functionality, including attachment, spread, proliferation, and differentiation. This survival capacity may have been facilitated by cultivating the HOBs in osteogenic media that were able to mitigate the oxidative stress and drive osteoblastic differentiation to maturation and matrix mineralization [24].

The study also suggests that NPs in OM are more biocompatible than MPs providing a useful guide to the design of specific surfaces using materials with nano topography to stimulate favorable cell responses, such as cell attachment, proliferation, and differentiation, or to avoid undesired side effects. The findings of this study may influence the selection of dental implants by dental practitioners, favoring nano-size over micro-size particles, which are related to lower inflammatory response in the peri-implant area.

## Limitations of the study

While particle concentration and size were easily controlled in this study, other factors such as surface topography and chemistry are extremely difficult to control since it is not possible to alter one factor without changing the other. This could be the major reason for different authors obtaining different results in osteoblast and titanium substrate cell–material interaction. This study’s findings could be a baseline for future research including animal studies and the long-term effect of titanium particles on the immune and bone cells.

## Conclusion

While HOBs exposed to either TiO_2_NPs or MPs at the tested sub-cytotoxic concentrations did not alter the cell phenotype, our findings demonstrated that TiO_2_MPs retarded osteogenic differentiation by inducing ROS generation, negatively influenced the level of ALP, OPG, and M-CSF and affected matrix mineralization density in comparison to TiO_2_NPs and control. Collectively, the study found TiO_2_NPs to be more biocompatible than TiO_2_MPs providing an insight into the capability of nanostructures in supporting osteoblast differentiation and its plausibility in biomedical applications.
